# Supplementary material for: Genomic islands targeting dusA in Vibrio species are distantly related to Salmonella Genomic Island 1 and mobilizable by IncC conjugative plasmids
Source: PLoS Genet. 2021 Aug 20;17(8):e1009669. doi: 10.1371/journal.pgen.1009669 (PMC8409611; doi:10.1371/journal.pgen.1009669)
Supplement: S1 Fig — Blastn and blastp atlases using either SGI1ΔIn104 (A) or IEVchUSA2 (B) as the reference. Coding sequences appear on the outermost circle in blue for the positive strand and red for the negative strand, with the oriT depicted as a grey arc. All other sequences are represented only according to their homology with the reference, with full opacity corresponding to 100% identity and gaps indicating identity below 60%. The order of the IEs in the atlases is indicated according to the color keys shown in the inset of panel B. (PDF) [file pgen.1009669.s001.pdf]

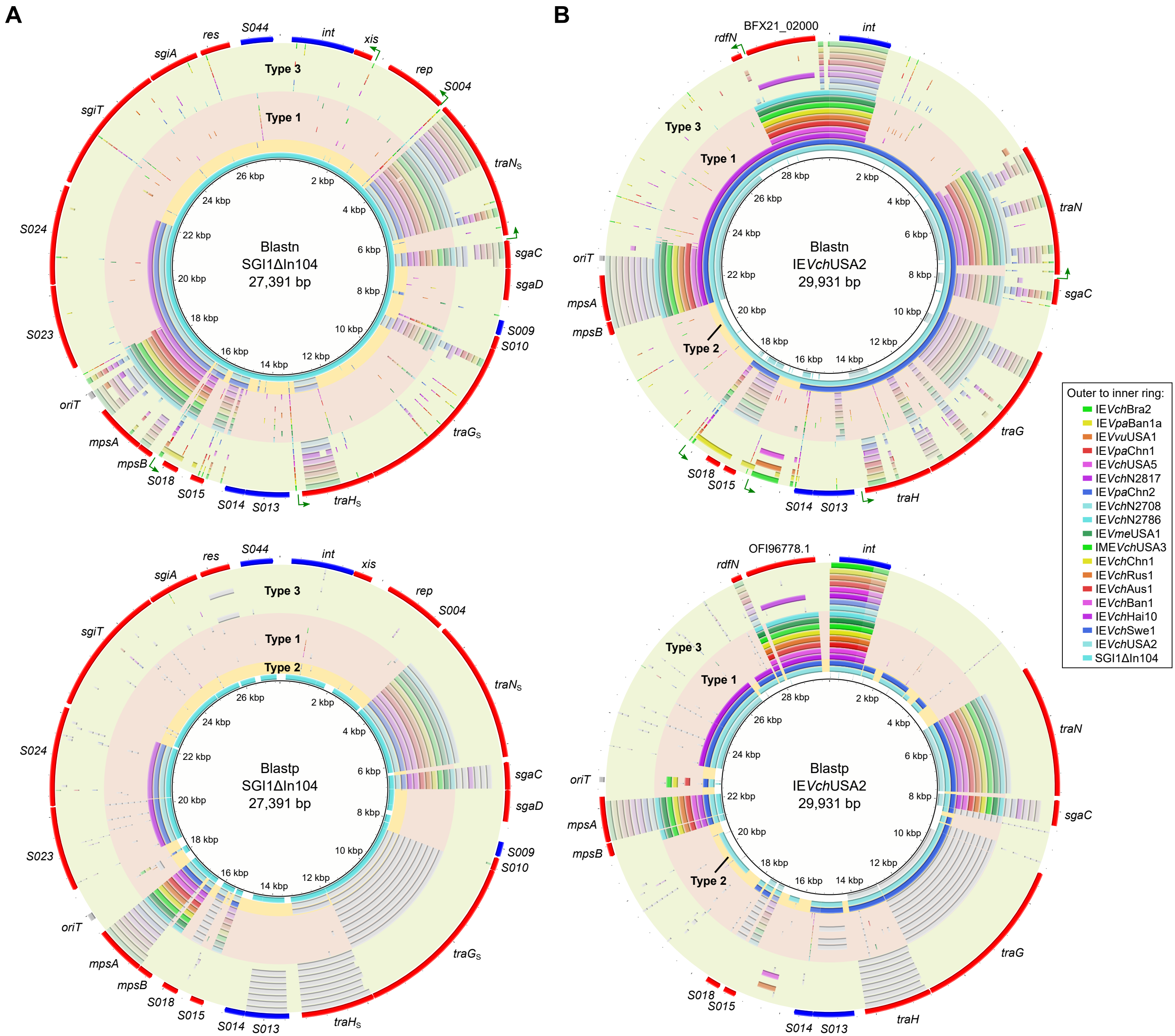
A
B
Blastn
SGI1ΔIn104
27,391 bp
Blastp
SGI1ΔIn104
27,391 bp
Blastn
IEVchUSA2
29,931 bp
Blastp
IEVchUSA2
29,931 bp
Type 1
Type 2
Type 3
int
xis
rep
S004
traN$_S$
sgaC
sgaD
S009
S010
traG$_S$
traH$_S$
S013
S014
S015
S018
mpsB
mpsA
oriT
S023
S024
sgiT
sgiA
res
S044
BFX21_02000
OFI96778.1
rdfN
traN
traG
traH
2 kbp
4 kbp
6 kbp
8 kbp
10 kbp
12 kbp
14 kbp
16 kbp
18 kbp
20 kbp
22 kbp
24 kbp
26 kbp
28 kbp
Outer to inner ring:
IEVchBra2
IEVpaBan1a
IEVvuUSA1
IEVpaChn1
IEVchUSA5
IEVchN2817
IEVpaChn2
IEVchN2708
IEVchN2786
IEVmeUSA1
IMEVchUSA3
IEVchChn1
IEVchRus1
IEVchAus1
IEVchBan1
IEVchHai10
IEVchSwe1
IEVchUSA2
SGI1ΔIn104
