# Supplementary figures and images for: Genomic islands targeting dusA in Vibrio species are distantly related to Salmonella Genomic Island 1 and mobilizable by IncC conjugative plasmids
Source: PLoS Genet. 2021 Aug 20;17(8):e1009669. doi: 10.1371/journal.pgen.1009669 (PMC8409611; doi:10.1371/journal.pgen.1009669)

**A**

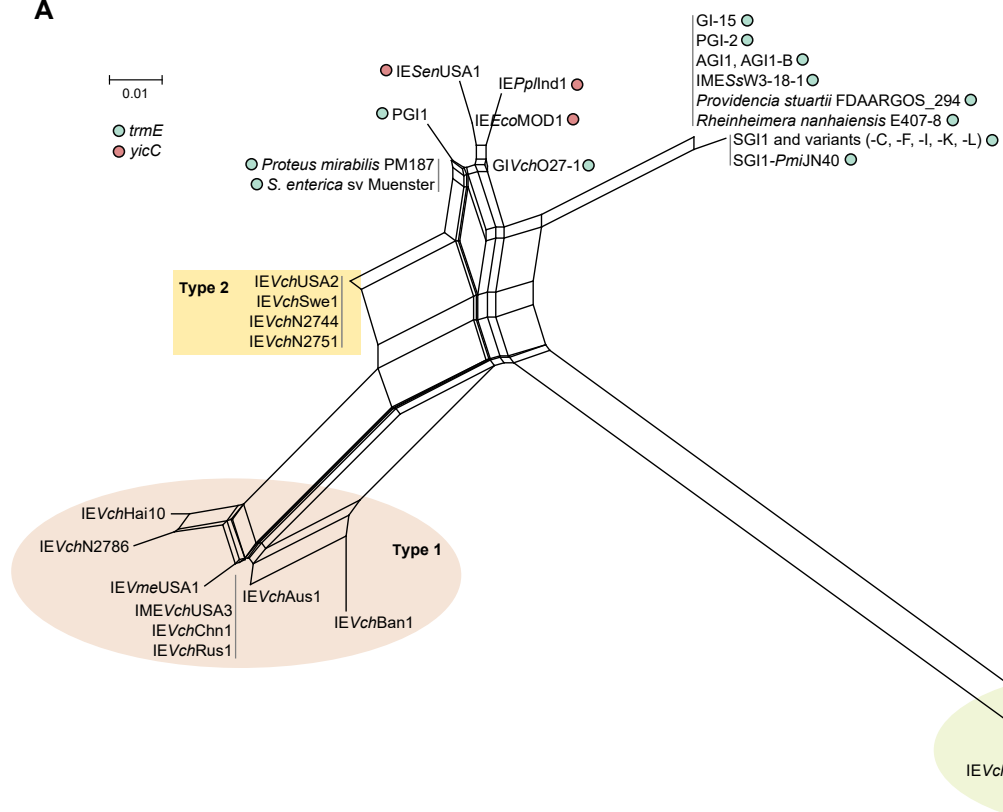

**B**

**Predicted *oriT* structure**

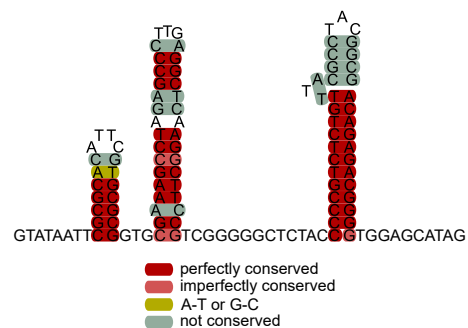

Supplement: S2 Fig — Each IE’s integration site and type are annotated. The sequence of canonical SGI1 (Genbank AF261825.2) was used as a reference to show the predicted secondary structure of all oriT sequences. Pairs can be perfectly conserved, imperfectly conserved (1/39 not conserved), not conserved (> 1/39), or an A-T or G-C pair only. In the latter case, the sequence is not conserved, but the predicted local secondary structure is. (PDF) [file pgen.1009669.s002.pdf]

**A**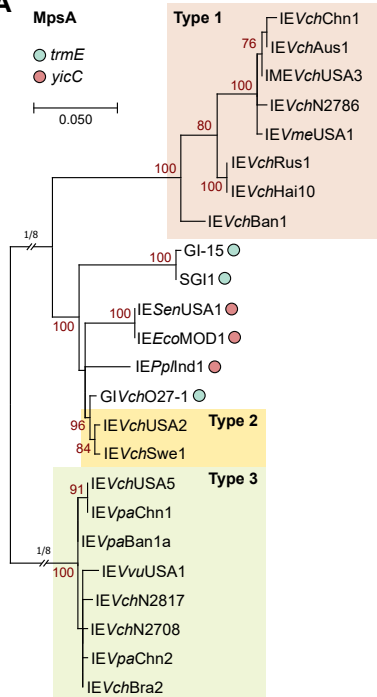**B**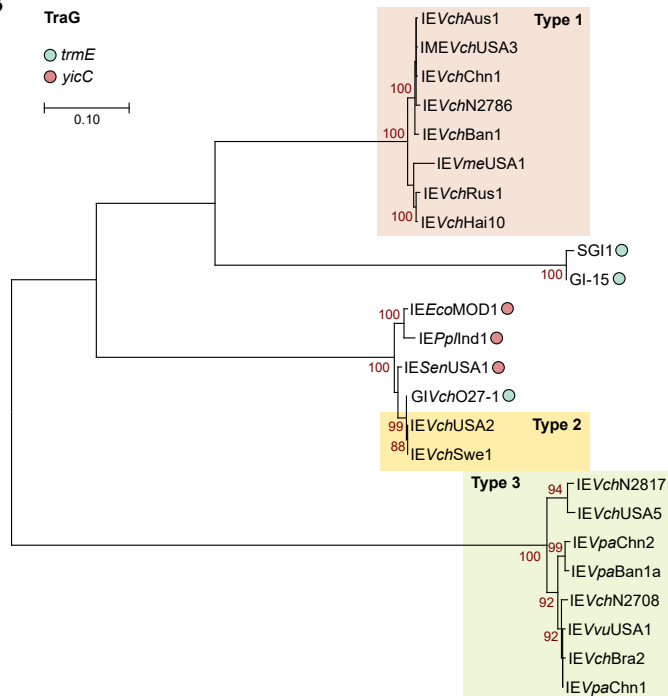**C**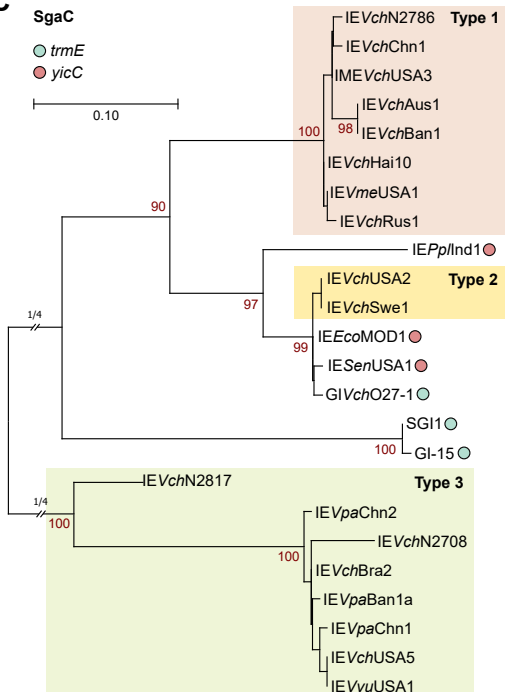**D**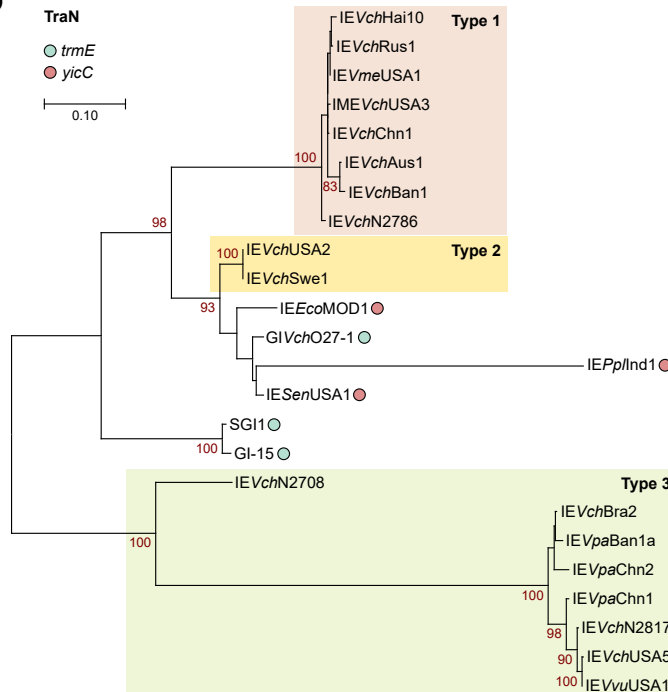

Supplement: S3 Fig — The trees for MpsA (A), TraG (B), SgaC (C) and TraN (D) proteins are drawn to scale, with branch lengths measured in the number of substitutions per site over 321, 1,145, 188, and 968 amino acid positions, respectively. For clarity, the lengths of the branches linking the two groups in panels A and C were artificially divided by 8 and 4, respectively. Taxa corresponding to IEs targeting trmE and yicC are shown by a light blue circle and a red circle, respectively. All other taxa correspond to dusA-specific IEs. Proteins accession numbers are provided in S1 Table and S2 Dataset. (PDF) [file pgen.1009669.s003.pdf]

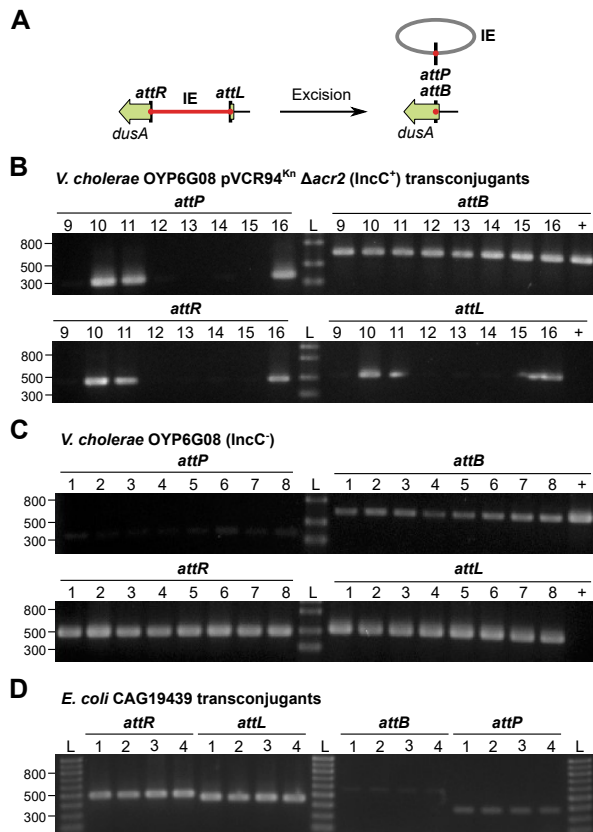

Supplement: S5 Fig — (A) Model of excision of IMEVchUSA3. (B and C) Detection of attB, attP, attL and attR sites by PCR in colonies of V. cholerae OYO6G08 bearing (lanes 9 to 16) or lacking (lanes 1 to 8) pVCR94Kn Δacr2. Control lanes: L, 1Kb Plus DNA ladder (Transgen Biotech); +, V. cholerae N16961 genomic DNA. (D) Detection of attB, attP, attL and attR sites by PCR in transconjugant colonies of E. coli CAG18439 (lanes 1 to 4). L, 100bp Plus II DNA Ladder (Transgen Biotech) (PDF) [file pgen.1009669.s005.pdf]
