# Supplementary material for: Genomic islands targeting dusA in Vibrio species are distantly related to Salmonella Genomic Island 1 and mobilizable by IncC conjugative plasmids
Source: PLoS Genet. 2021 Aug 20;17(8):e1009669. doi: 10.1371/journal.pgen.1009669 (PMC8409611; doi:10.1371/journal.pgen.1009669)
Supplement: S4 Fig — Promoter sequences are grouped based on the function of the expressed genes as follows: (A) RDFs; (B) mating pair stabilization; (C) mating pair formation and stabilization; (D) unknown. AcaCD binding sites are shown in green. Logo sequences and p-values were generated by MAST [51]. Known transcription start sites are shown in blue [17,22]. Predicted Shine-Dalgarno sequences are shown in pink. The initiation start codon is shown in bold letters. (PDF) [file pgen.1009669.s004.pdf]

A

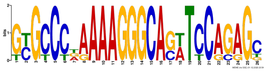

	Sequence	*p*-value
SGI1 *xis*	TTCGCGCCCTAAAAGGGCAGATCCAGAGCCGAGGATTGGTAGCTGTCTCTCATAAACTAGGTCTATCAATAACTGGTGTGTAACGGGTAGGCTGATG	4.2E-10
GI-15 *xis*	TTCGCGCCCTAAAAGGGCAGATCCAGAGCCGAGGATCTGAAACCTGTGTTGGTAGACTAAACCAG(22)AGATATGACTATGCAAGAGACAGAGTTG	4.2E-10
GI*Vch*O27-1 *xis*	GAGTTGTCCAAAAAGGGCACTTTCAGAGCGGAGGTTTCTCAACGGGCACCCATAAAATTGCAGTC(6)AGGGTTGCCAATGAAGTAGGTATGCGATG	6.8E-08
IE*Eco*MOD1 *rdfM*	TTTGCGCCCGAAAAGGGCACTTCCAGAGGTGAGGTTTTTAGACGCCACCCAGCAAAATTGCCCCTGTGATCAAATTCATCAGGGGAACCTATG	6.8E-12
IME*Vch*USA3 *rdfN*	GTGGTGCCCGAAAAGGGCAGTTCCAGAGCCGATGATTTGCAGTGTTGAACCTTAGGATCGTTGGAAATTGTTGAGAATGATTGAGGATAGCTATG	4.1E-11
IE*Vch*USA5 *rdfN*	TAAGTGCCTAGAAAGGGCAGATCCGCGGTATGTCTCGCATGTTGAAATAAACGAAAATGATGGCGTTTAGTTGAGGAATG	6.5E-08

B

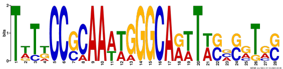

	Sequence	*p*-value
SGI1 *traN*$_S$	CGTATGCCGCAAAAGGGCAAATAGCGATGCTAATTTTTATGAGAGAGCGACATAGCATTATCCAAACTAAAAAGCTGGAGAAATGCTATG	6.4E-06
GI-15 *traN*	CGTATGCCGCAAAAGGGCAAATAGCGATGCTAATTTTTATGAGAGAGCGACATAGCATTATCCAAACTAAAAAGCTGGAGAAATGCTATG	6.4E-06
GI*Vch*O27-1 *traN*	GATTTTCCGCAATTGGGCAGTTTGGCGTAGGAGGTTTATTACTTCAGCCGAATAGCATAGCTCTCAAGATTTTATTGAGAGCTATGCTATG	5.4E-06
IE*Eco*MOD1 *traN*	GATTTTCCGCAATTGGGCAGTTTCGCGTAGGAGGTTTATTACTTCAGCCGAATAGCATGGTTCTCAAGATTTTATTGAGAGCCATGCTATG	3.7E-06
IME*Vch*USA3 *traN*	TGTTTTCCCAAAATGGGCAGTTTCACCGCGTAGGTTTTGGTGGGTGAGCTTTTAACATGATTTTCTAACGCTTTTATTTCCGGAAATCATGTGATG	2.3E-08
IE*Vch*USA5 *traN*	AATGCACCCCAAATAGGCAATTACAGGTGCGTAAGAATTTTAAATACTGAAATAGCATGAACCATTGAAAATGGAGGTATCATCTATG	2.8E-08

C

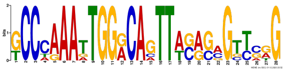

	Sequence	*p*-value
SGI1 *traHG*$_S$	GGACTGCCCAAATTGGACAGTTTGGGAGTTCGGTTTGTACTCATTTGCTCGGTAGGCTTTCGGGTGACACGAAACCTATTGGAGCAACAGTATG	8.9E-09
GI-15 *traHG*	GGACTGCCCAAATTGGACAGTTTGGCAGTTCGGTTTGTACTCATTTGCTCGGTAGGCTTTCGGGTGACACGAAACCTATTGGAGCAACAGTATG	7.9E-09
GI*Vch*O27-1 *traHG*	AGGATGCCCAAAATGGACACTTACAGCGTTCGGTTTGCCGATTATCGGTGGTTACTCTGGAGCCAACGACTATCCGCAAATTGAGGTTGAAATG	2.5E-10
IE*Eco*MOD1 *traG*	TAAACGCCAAAATTGGGCAGTTACAGAGTCGTGGTAAAGAGGATTGAGCGAATAGGCTATCTCCAAATTTCGGGAGAATAAGCCTATG	5.6E-11
IME*Vch*USA3 *traG*	ATTATTCCTGAATTGGGCAGTTACACGGGCGAGGTTTGAAGCCAAATTGCGATAGGATGAACGGATCAAGTCGGGAGTATTTGTCTATG	8.2E-07
IE*Vch*USA5 *traHG*	ATTGCTCCCAAAGTGGGCAATTAGAGCGCTAAGGAATAGCGCTAATGATTGAGAAAATAGAAACTCAAATTCGGATGGAGAGTTTCTATTATG	6.0E-07

D

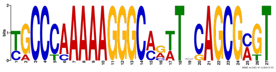

	Sequence	*p*-value
SGI1 *S018*	AATGTGCCCAAAAAGGGCCAATACAGCGCGTGTCGATTCAGAAATGAGCTTGTTAAATTTGAATC(110)AATTCCAACTAATTGGAGGTGTTTATG	2.8E-12
GI-15 *S018*	ATTGTGCCCAAAAAGGGCCAATACAGCGCGTGTCGATTCAGAACTGAGCTGTTAAATTTGAATTA(109)AATTCCATCTAATTGGAGGTGTTTATG	4.7E-12
GI*Vch*O27-1 *S018*	TATGCGCCCAAAAAGGGCAGTTCCAGCGAGTATCCCCTGACGCCTTGGCTGTTACAGTGTTCACA(101)CCTCAAAGAAATTTGAGGTGTTGTATG	3.2E-13
IE*Eco*MOD1 *S018*	ACGATGCCCAAAAAGGGCAGTTCCAGCGAGTATCCTCTGACGTATTGCTTGTTAAAGTGTTCTCA(101)CCTCAAAGAAATTTGAGGTGTTGTATG	1.9E-08
IME*Vch*USA3 *S018*	CGTTTACCCCAAAAGGGCACTTTCAGCGCGTTCCATCACATCCCATCTTTTATAAACTATCCACA(101)CCCTTCAACTTTTTGGAGGTGTCTATG	1.0E-07
IE*Vch*USA5 *S018*	AGTGCGCCTAAAAAGGGCAGTTGGAGCGATTTGTCTAAGTAGAACAGCCGGTTATCTTGGAAGTT(104)CCCTCAAAAATATGAGGTGAAGCTATG	1.4E-06
