# Supplementary material for: Genomic islands targeting dusA in Vibrio species are distantly related to Salmonella Genomic Island 1 and mobilizable by IncC conjugative plasmids
Source: PLoS Genet. 2021 Aug 20;17(8):e1009669. doi: 10.1371/journal.pgen.1009669 (PMC8409611; doi:10.1371/journal.pgen.1009669)
Supplement: Attachment — Submitted filename: Response to reviewers.docx [file pgen.1009669.s012.docx]

Reviewer #1: This manuscript describes novel genomic islands related to SGI1, identified in other target sites and other bacterial species than reported for SGI1 since 20 years. The islands were functionally assessed for their mobility in relation with IncC plasmids required for their mobilization. The authors propose an interesting evolutionary model based on the data of this study that may have resulted in the epidemic SGI1 and its variants. The manuscript is clearly written and I have only minor comments or questions:

1. The authors have focused their study on mobilization genes of the new islands identified. Do these islands carry genes of medical importance such as antimicrobial resistance genes or other genes that may confer an advantage to the bacterial pathogen such as virulence genes? If so the authors may consider including this information in the text.

We have added a more descriptive characterization of the gene content of the IEs in a new section “Variable features found in the *dusA*- and *yicC*-specific IEs”. We also added a new S1 Dataset file that contains the description of all features predicted in each of the IEs reported in this manuscript.

2. For SGI1 besides trmE another integration site has been reported (Doublet et al., PLoS One. 2008 Apr 30;3(4):e2060. doi: 10.1371/journal.pone.0002060). In some genomes SGI1 relatives occur in this integration site.
This information was already stated at the beginning of the discussion. However, we slightly modified the text to be more accurate (lines 425-426).

3. Lines 75 and 117, SGI1 is presented as one of the most "atypical" and most studied IMEs. The use of "atypical" in these lines is not clear to me. Why is SGI1 atypical?

We thank this reviewer for this remark. We agree that the word “atypical” was poorly chosen and confusing. All instances of “atypical” have been removed from the introduction.

Reviewer #2: Overall this is potentially a solid study that is of interest because it extends the group of IME that use the unique IncC plasmid-integrative mobilizable element (IME) relationship studied to date mainly in the case of the IME SGI1 and variants of SGI1. However, there are problems with presentation - it’s jumbled - that does not help readers follow the stories presented. Thinking through the findings of the work to consider what to include, to find a more logical order, and improvement of the Figures would be very beneficial.

We thank this reviewer for bringing this issue to our attention. The first two sections of the results have been reorganized and rewritten for clarity. The figures have also been reorganized for that purpose.

This study identifies a series of integrative mobilizable elements (IME) that include genes that are homologues of some of the important genes found in SGI1 (and relatives), the best studied of the bacterial IME. In SGI1, these genes are associated with mobility and dependence on an IncA or IncC plasmid for intercellular transfer. The novel IME, mostly found in Vibrio genomes, are not found in the same location as SGI1 because they encode different Int to that used by SGI1 and hence integrate at 2 different locations, dus for the Vibrio set and yicC for the others. This is not particularly surprising as groups of Int are known to be specific for a specific site and the sites are ones known to be recognised by specific groups of Int.
This aspect of the work is not well analysed or well organised or presented. The analysis is minimal and does not include critical information that is standard for this type of work such as accession numbers for the sequences used in phylogenies - they can be listed in the Fig legend. The Figures are not of publication quality and Fig. 2 is not the best to complement or inform the text.

As stated above, this part of the manuscript has been reorganized. We have also improved access to data used in the manuscript and figures. All accession numbers of proteins used in the phylogenetic analyses are now explicitly provided for each IE in S1 Table. Figure 2 has been moved to supplemental data and replaced with new panels in Figures 2 and 3 that describe the ranges of identities for each core protein in the different types of IEs. Figure 1 has been enhanced for clarity (colors and organization of the IEs). Two new supplemental datasets (S1 and S2) have been added. S1 dataset describes all features predicted in each IE reported in this manuscript. S2 dataset provides the clusters of core proteins used in Figures 2C and 3B.

The study also addresses some initial basic questions relating to the properties of one of the IME (called MGIVchUSA3) that targets dus, including showing that:
1. The IME is excised in the presence of an IncC plasmid and this is dependent on rdf, thus identifying the gene for the recombination directionality factor (rdf).
2. Only 2 of 4 potential AcaCD binding sites identified are AcaDC responsive – one is upstream of rdf. So, this IME may not have been the best choice to study.

We disagree with this opinion. On the contrary, IMEVchUSA3 is an excellent choice. First, it shows the limitations of AcaCD boxes prediction. The AcaCD box detection matrix was build using AcaCD-responsive promoters in “ideal” elements such as pVCR94 and SGI1 (PMID 25340549). We currently do not know the extent of polymorphism allowed in AcaCD binding sites. Second, IMEVchUSA3 also shows that despite a few imperfections (*traH* and *sgaD* missing, the promoters of *traN* and *S018* seemingly inactive), the IME remains mobile as essential functions have been preserved. In conclusion, this suggests that most if not all of the IEs we detected are functional.

3. SgaC, the AcaC homologue encoded by the IME, can substitute for AcaC (interacting successfully with AcaD) as there is no sgaD gene in this IME
4. IncC plasmid-assisted transfer.
5. IME that use the 5’-end of dus supply a replacement 5’-end and a promoter.
However, a well annotated GenBank entry (a TPA is possible if the sequence belongs to others) for the IME used is needed to follow this work but I could not find reference to one. This MUST be supplied.

Indeed, the sequence of *V. cholerae* OYP6G08 that contains IME*Vch*USA3 belongs to Yann Boucher. Although we are not skeptical of the necessity for a new accession number for IME*Vch*USA3 as we provided ample details regarding its exact position in the genome in S1 Table, we submitted a TPA request via Genbank’s BankIt on July 13, 2021 (see attached Genbank’s confirmation e-mail). Unfortunately, to date, the submission has not been processed despite multiple requests. Since the sequence is already publicly available via Genbank NZ_NMSY01000009, this remark CANNOT be a valid reason for denying or delaying the publication of this work.

Finally, a highly speculative model for the evolution of the IncC-mobilizable group is presented.

We acknowledge that the model for the evolution of the IncC-mobilizable IE we presented in Figure 7 is speculative. It is presented as such in this manuscript. However, based on the current knowledge, sequences of IEs available in the databases, and our analyses, it is currently the most robust and reasonable hypothesis until proven otherwise.

Major comments:
1. Use of MGI and GI. Why call an IME MGI? Why use GI? GI is a very generic term that be used for both IE and Tn as well as diverged regions replaced by homologous recombination. IE must integrate using a site-specific recombinase. In 2021, it’s time to be more accurate.

IE is rarely used in the literature, and the term IME can be easily confused with ICE, especially in naming schemes in papers dealing with both. Historically, our group has characterized the biology of this type of element since 2010 (PMID: 20807202, 22923590, 23204461) before the publication of the IME review by Bellanger et al. in 2014 (PMID 24372381). In the following years, we discovered other IMEs (PMID: 27435459, 32848007), and for the sake of consistency and simplicity, we kept using MGI and GI in our naming scheme. Nevertheless, to quote Shakespeare, “What's in a name? That which we call a rose by any other name would smell as sweet”. We aim to understand the biology and evolution of these elements, not to bicker about made-up nomenclatures. Hence, we replaced GI and MGI with IE and IME, respectively, throughout the manuscript and figures, except for the GIs and MGIs previously described in the literature.

Also, Kn for kanamycin! There are 2 accepted abbreviations Km or KAN. One of these should be used.

The abbreviation Kn for Kanamycin is defined in line 541 and Table 2. Kn is used unambiguously throughout the text. Changing Kn for Km or KAN in the text will only add confusion for the previously published kanamycin-resistant plasmid construct pVCR94^Kn^.

2. The Introduction could be a bit more compact.

We have reduced the size of the introduction.

3. There are too many references (73 currently). Those that are not directly relevant are not needed, e.g. ref. 8, this information is reviewed in refs. 12, 13 and 14, and should be culled extensively.

The number of references is down from 73 to 66.

4. Figures are not of publication quality, particularly Figs. 1 and 2 which are blurred and have font size verging on unreadable. In Figure 1, the various blue shades are not easily distinguished.

Unfortunately, authors have little control over the quality of the figures in the PDF file sent for review regardless of the quality of the files provided to the Editorial Manager platform used by PLOS journals. However, high-resolution images of the figures could be downloaded by clicking on the link at the top of each figure in the PDF file. We have modified Fig 1 nonetheless to enhance the contrast between blue shades. Other enhancements have also been made (see remark 5).

5. Splitting Fig. 1 to show more clearly the features of SGI1 (for the Introduction) and the IME used experimentally (for the Results) would be helpful. The yicC group could also be separated.

The different IME types are now clearly indicated in Fig 1. IMEs are separated according to their integration sites and types for the *dusA*-specific IMEs. The grey shading has been removed.

6. Re-organisation of the components of other Figures to better group items related to the same issue and put them in an order more consistent with the text is needed.

Figures have been reorganized for clarity.

7. Fig. legends should describe the Figure not the Methods used. Please fix where relevant.

The legends of the figures have been verified and methods have been removed where relevant.

8. lines 106-7, Other IE, particularly ICE, do carry rep genes and replicate.

We are perfectly aware of ICE replication. We have published a manuscript about this topic a few years ago in PLOS Genetics (PMID 26061412). Besides, ICEs usually rely on the relaxase and oriT to replicate, not on a genuine separated replicon. Actinomycete ICEs (AICEs) are the exception because they transfer as double-stranded DNA. Nevertheless, this is out of topic here. The text is referring to IMEs, not IEs in general, AICEs or ICEs. To the best of our knowledge, SGI1 is the only IME that undergoes active replication via a dedicated replicon, as shown by us and others.

9. line 132, Is “resembling” the right word here. In the Introduction it’s said they don’t have to resemble. Perhaps “sharing features with”.

We have rephrased using “distantly related to”.

10. A simple Table showing the basic features of the IME shown in Fig. 1, e.g size, organism, and where their sequences can be found (Accession numbers) is needed in the main body of the article.

This is quite redundant with S1 Table. Nevertheless, we added a simplified version, new Table 1, in the body of the article.

11. Please state the consensus used to predict AcaCD binding sites. The legend to Figure 1 must clearly state that the marked AcaCD binding sites are potential; you later show 2 are not responsive.

The matrix for detecting AcaCD binding sites using MAST or FIMO is now provided in S1 Matrix and indicated in lines 674-675. The legend of Figure 1 now states explicitly that AcaCD binding sites are “potential”.

12. what % identity is indicated by the grey shading in Fig. 1; is it identity of DNA or proteins. Please add this information to the legend.

The grey shading has been removed from Fig 1 and replaced with panels showing heatmaps of similarities for proteins conserved in the different IME types in Figs 2 and 3.

13. Some % identities (or ranges) for the similar proteins are needed in the text.

This information is now provided as heatmaps (see remark 12) and described in the text.

14. Line 115, “unrelated” Surely not, they are both tyrosine recombinases.

First, line 115 is not about recombinases. Perhaps this reviewer meant line 155. Second, we clearly stated, “Consistent with the change of integration site, the respective ***int* genes** of SGI1 and the *dusA*- and *yicC*-specific GIs are unrelated.” Indeed, blastn searches failed to find any significant similarity between these genes. Integrase genes are known to be fast-evolving. Although the proteins all contain the same C-terminal Phage_integrase (PF00589) catalytic domain, the genes bear no similarity with one another. Besides, the N-terminal halves of these three tyrosine recombinases contain different Pfam domains, as shown below. Furthermore, the proteins themselves share no significant similarities, as shown in the heatmap presented in Figure 2C.

SGI1 Int (405 aa residues, *trmE*)

| **Family** | **Description** | **Clan** | **Envelope** | | **Alignment** | | **HMM** | | **E-value** |
| --- | --- | --- | --- | --- | --- | --- | --- | --- | --- |
|  |  |  | **Start** | **End** | **Start** | **End** | **From** | **To** |  |
| [Phage_int_SAM_5](http://pfam.xfam.org/family/PF13102.6) | Phage integrase SAM-like domain | [CL0469](http://pfam.xfam.org/clan/CL0469) | 94 | 209 | 94 | 193 | 1 | **95** | 6.8e-24 |
| [Phage_integrase](http://pfam.xfam.org/family/PF00589.22) | Phage integrase family | [CL0382](http://pfam.xfam.org/clan/CL0382) | 206 | 369 | 207 | 366 | **2** | **169** | 3.0e-18 |
| [Arm-DNA-bind_5](http://pfam.xfam.org/family/PF17293.2) | Arm DNA-binding domain | [CL0081](http://pfam.xfam.org/clan/CL0081) | 3 | 95 | 4 | 68 | **2** | **61** | 1.3e-05 |

IME*Vch*USA3 Int (366 aa residues, *dusA*)

| **Family** | **Description** | **Clan** | **Envelope** | | **Alignment** | | **HMM** | | **E-value** |
| --- | --- | --- | --- | --- | --- | --- | --- | --- | --- |
|  |  |  | **Start** | **End** | **Start** | **End** | **From** | **To** |  |
| [Phage_integrase](http://pfam.xfam.org/family/PF00589.22) | Phage integrase family | [CL0382](http://pfam.xfam.org/clan/CL0382) | 184 | 346 | 199 | 344 | **16** | **170** | 1.3e-16 |

IE*Sen*USA1 Int (403 aa residues, *yicC*)

| **Family** | **Description** | **Entry type** | **Clan** | **Envelope** | | **Alignment** | | **HMM** | | **E-value** |
| --- | --- | --- | --- | --- | --- | --- | --- | --- | --- | --- |
|  |  |  |  | **Start** | **End** | **Start** | **End** | **From** | **To** |  |
| [Arm-DNA-bind_3](http://pfam.xfam.org/family/PF13356.6) | Arm DNA-binding domain | Domain | [CL0081](http://pfam.xfam.org/clan/CL0081) | 3 | 88 | 4 | 87 | **2** | **73** | 1.9e-15 |
| [Phage_integrase](http://pfam.xfam.org/family/PF00589.22) | Phage integrase family | Domain | [CL0382](http://pfam.xfam.org/clan/CL0382) | 201 | 383 | 203 | 354 | **3** | **141** | 4.2e-13 |

15. Line 165, which 5? Please state here or explain how to find them in the Fig.

These 5 elements were easy to spot in Fig 1 as they are the only ones with an orange ORF labeled “*repA*”. Regardless, they are now also listed in the text line 244.

16.lines 176-189, one name (GIVchBan1) for 2 IE – that’s not sensible. It’s well known that a further IE can integrate where one is already present. So, each should have a name.

We now have two different names for these two accreted IEs, IE*Vpa*Ban1a and IE*Vpa*Ban1b.

17. lines 182-233. This section is close to impossible to follow. It needs a vast improvement if any reader is to follow it. Which genes are present is in Fig..1. So, the BRIG alignments in Figure 2 are not helpful (may be OK as supplementary). Some detail as the to % identities of genes/proteins within specific groups is essential here. This can easily be tabulated and this Table is needed in this section. Only the phylogeny for MpsA-TraG-SgaC-TraN (and the supplementary phylogenies for individual genes in Fig. S2) are needed here. The Int proteins were discussed in the previous section.

As indicated above, this section has been deeply reorganized. We have taken into account all the comments of this reviewer to improve readability.

18. VchN2708 is the outlier to type 3 in 2A but Vch2719 is the only outlier in S2. Are they labelled correctly?

This reviewer probably meant VchN2708 in Fig 3A (not 2A) and VchN2817 (not Vch2719) in Fig S2C and S2D. Yes, both are correctly labeled in the respective trees. VchN2708 is an outlier in 3A due to the higher weight of TraN (~968 aa residues) compared to SgaC (~188 aa residues) in the tree of concatenated proteins. The divergence of TraN of GIVchN2708 in S2D (now S3D) outweighs the divergence of SgaC of VchN2817 in S2C (now S3C) due to protein size difference.

19. Lines 266-293, Why was this done with a de-repressed IncC plasmid? This section is not needed and should be removed. The information and Figure could be Supplementary.

Interspecific conjugative transfer of pVCR94 between *V. cholerae* and *E. coli* is inefficient. To increase the odds of getting transconjugants, we used the derepressed plasmid (also see the response to remark 21). We respectfully disagree with the statement regarding the section 266-293 (now 298-314). The results presented in this section show that the IME excises in response to the IncC plasmid in *V. cholerae* and demonstrate the interspecific transfer of the IME. Most of the corresponding text was retained in a condensed format, and Fig 5 is now S5 Fig.

20. In Figs., 5A and 6D, why is attR on the left and attL on the right? This is confusing. In addition, usually L is near the int gene. It’s easy to fix.

For the elements integrated at *dusA*, we used the nomenclature of Farrugia *et al.* (2015, PMID 25883135) to label *attL* and *attR*, with *attR* near the *int* gene. These authors have precedence on *dusA*-specific genomic islands. The reason for this annotation is now indicated in the legend of Fig 1.

Please convert the bar graphs to scatter plots so that the number and range of values determined can be seen.

The replicate values are now plotted where appropriate. Bars have been conserved for clarity.

21. Line 304, so the IME transfer frequency is higher when assisted by the standard plasmid compared to with the de-repressed equivalent? Please explain.

The derepressed plasmid was used to enhance the interspecific conjugative transfer of both the plasmid and IME from *V. cholerae* to *E. coli*. Intraspecific transfer of pVCR94 (*E. coli* to *E. coli* or *V. cholerae* to *V. cholerae*) always occurs at a much higher rate, even using a non-derepressed plasmid. This phenomenon could result from the action of a species-specific restriction-modification system. This observation was already reported elsewhere (PMID: 24567731).

22. I’ll stop here and say, a manuscript should be better prepared before submission. It is not a reviewer’s job to do this much work.

We appreciate this reviewer’s attention to detail and are grateful for her or his comments. Nevertheless, a fair number were only minor and did not deserve to be in the Major comments section. This last remark (22) is damaging, demeaning, and uncalled-for.

Minor comments:
Lines 86-7, add florfenicol.

Florfenicol was added line 88.

Line 97, Isn’t the HKL group defined in ref. 14? “Seem to”? There are sequences.

All reference to the HKL group was removed as unnecessary in this context.

Line 101, add the hyphen to 3’ end here and elsewhere and 5’ end elsewhere.

The hyphen in this context does not seem appropriate. 3’ and 5’ are not the first part of a compound adjective. We will let the publisher decide.

What is meant be the text of lines 118-120. Please clarify. Are you simply saying you used protein sequences in your searches as is quite standard?

This section was rewritten to be more explanative.

Line 194-5, do the authors of ref. 25 really call MspA a tyrosine recombinase or just allude to similarities?

This reviewer seems to refer to lines 104-5. Taken from the abstract of ref 25 (now 19) “The protein structure predictions imply that MpsA belongs to the lambda tyrosine recombinase family” and the discussion section “Phyre2 modeling confirmed that MpsA is related to the Tyr-recombinase family, as the best templates with known 3D-structure were XerH (Fold library id.: c5jjvA) and Cre recombinase (Fold library id.: c1ma7A) with 15–13% sequence identity, 91–94% coverage and 100% confidence.” However, we agree that experimental evidence is missing. Hence, we modified the sentence accordingly (lines 105-106).
